# A common polymorphism in *SNCA* is associated with accelerated motor decline in *GBA*-Parkinson’s disease

**DOI:** 10.1136/jnnp-2019-322210

**Published:** 2020-04-02

**Authors:** Thomas B Stoker, Marta Camacho, Sophie Winder-Rhodes, Ganqiang Liu, Clemens R Scherzer, Thomas Foltynie, Roger A Barker, Caroline H Williams-Gray

**Affiliations:** 1 John van Geest Centre for Brain Repair, Department of Clinical Neurosciences, University of Cambridge, Cambridge, UK; 2 Wellcome Trust ‑ Medical Research Council Stem Cell Institute, University of Cambridge, Cambridge, UK; 3 School of Medicine, Sun Yat-Sen University, Guangzhou, Guangdong, China; 4 Advanced Center for Parkinson's Disease Research, Harvard Medical School, Brigham and Women's Hospital, Boston, Massachusetts, USA; 5 Precision Neurology Program, Harvard Medical School, Brigham and Women's Hospital, Boston, Massachusetts, USA; 6 Department of Clinical and Movement Neurosciences, UCL Institute of Neurology, London, UK

**Keywords:** Parkinson's disease, neurogenetics, movement disorders, Lewy body

A growing number of genetic susceptibility factors have been identified for Parkinson’s disease (PD). The combination of inherited risk variants is likely to affect not only risk of developing PD but also its clinical course. Variants in the *GBA* gene are particularly common, being found in approximately 5% to 10% of patients, and they lead to more rapid disease progression.^1^ However, the effect of concomitant genetic risk factors on disease course in *GBA*-PD is not known.

The aggregation of α-synuclein, encoded by the *SNCA* gene, is central to the pathogenesis of PD. The *SNCA* rs356219 A/G polymorphism alters the risk of developing PD, with homozygotes for guanine (G/G) having an increased risk compared with carriers of an adenine (G/A or A/A) at this locus.^2^ The relationship between glucocerebrosidase (the enzyme encoded by the *GBA* gene) and α-synuclein is complex. These proteins have been shown to interact directly in vitro, as well as to influence the intracellular levels and processing of each other, potentially in a bidirectional feedback loop.^3 4^ Interestingly, a recent genome-wide association study found that the presence of this *SNCA* polymorphism was associated with an increased likelihood of developing PD in *GBA* mutation carriers.^5^ We therefore hypothesised that the presence of the *SNCA* rs356219 polymorphism would accelerate the clinical course of *GBA* variant-associated PD. Here, we report on the effect of this *SNCA* polymorphism on clinical outcomes within the *GBA*-PD population.

Longitudinal data from *GBA*-variant carriers were analysed from the community-based ‘Cambridgeshire Incidence of Parkinson’s disease from General Practice to Neurologist’ (CamPaIGN) cohort (n=142).^6^ This study was approved by the local ethics committee and written informed consent was obtained from all subjects. Newly diagnosed patients were followed up with assessments every 2 years for up to 18 years. Time to development of dementia (defined as Mini-Mental State Examination (MMSE) score of 24 or less, with fulfilment of Diagnostic and Statistical Manual of Mental Disorders IV criteria), progression to postural instability (Hoehn and Yahr stage three (HY3)) and death were determined.

Sequencing of the *GBA* gene was carried out in 114 patients in the CamPaIGN cohort, as described here.^7^ A further 16 patients underwent targeted genetic screening for common *GBA* variants using the Illumina Multi-Ethnic Genotyping Array (MEGA) chip. Genetic analysis of the *SNCA* rs356219 polymorphism had also been previously performed in 124 patients from the cohort.^2^ *GBA* variants were identified in 20 patients, which were included in survival analyses. Of these, 7 carried the high-risk (G/G) *SNCA* genotype, with 13 carrying low-risk (G/A or A/A) genotypes. There were no differences in age at diagnosis nor in baseline assessments including total and part III Unified Parkinson’s Disease Rating Scale scores, Hoehn and Yahr scores, MMSE scores or levodopa equivalent doses between these *SNCA* genotype groups. All carriers of the G/G genotype were male, compared with 53.8% in the low-risk group (p=0.03) (online supplementary table 1).

10.1136/jnnp-2019-322210.supp1 Supplementary data

**Figure 1:**
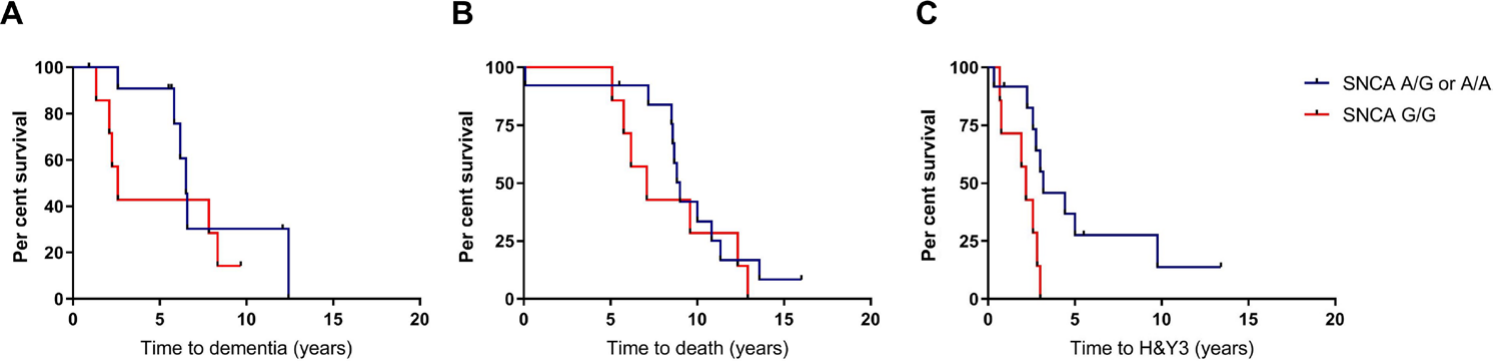
Survival analyses in *GBA*-PD patients comparing those with high-risk and low-risk variants in the *SNCA* rs356219 polymorphism. Kaplan-Meier curves for time to (A) dementia, (B) death and (C) postural instability. A, adenine, G, guanine; H&Y3, Hoehn and Yahr stage three; PD, Parkinson’s disease.

Survival analyses for the aforementioned outcome measures were performed, with statistical significance determined using log-rank tests. P-values were adjusted for multiple comparisons using the Bonferroni method. Among carriers of *GBA* variants, there were no differences between the high-risk and low-risk *SNCA* groups for time to dementia (p=0.29, adjusted p=0.86) or death (p=0.43, adjusted p=1.28) (figure 1A, B). Progression to HY3, however, was significantly faster in the G/G *SNCA* group (p=0.02, adjusted p=0.07), with mean time to development of postural instability 2.0 years (95% CI 1.3 to 2.7) compared with 4.9 years (95% CI 2.5 to 7.3) in A carriers (figure 1C). All G/G carriers reached HY3 within 3 years of diagnosis.

To account for potential confounders, time to HY3 was also compared using a Cox regression model controlling for sex and age at diagnosis. The G/G *SNCA* genotype was associated with a HR for progression to HY3 of 3.8 (95% CI 0.9 to 16.1), p=0.07, adjusted p=0.21) when controlling for these confounders. Because all the *SNCA* G/G carriers were male, we also performed a survival analysis in which only male subjects were included, to further control for sex as a potential confounder. An increased risk of progression to HY3 was again observed in G/G carriers in comparison to A carriers (p=0.036, adjusted p=0.11; online supplementary figure 1). *SNCA* genotype had no effect on progression to HY3 in non-carriers of *GBA* variants (n=85) in the CamPaIGN cohort (p=0.88, adjusted p=2.65; online supplementary figure 2).

This cohort contained four patients with the non-coding c. 762–18 T>A variant (online supplementary table 2). While this has been reported to be a potential risk factor for PD,^8^ its importance is not clear. We therefore also performed analysis after exclusion of these patients and found that progression to HY3 was greater in the G/G carrier group, with a HR of 5.3 (95% CI 1.1 to 26.2; p=0.041, adjusted p=0.12).

To our knowledge, no previous studies have investigated the influence of concomitant genetic risk factors on the progression of *GBA*-PD. Here we report that the *SNCA* rs356219 polymorphism significantly affects motor progression in *GBA*-PD, with the G/G genotype associated with a particularly aggressive disease course. This effect was not observed in patients with PD without *GBA* variants, suggesting that it was relatively specific to *GBA*-PD.

This new study therefore raises the interesting possibility that *GBA* variants and the G/G *SNCA* rs356219 polymorphism act synergistically to accelerate pathology and clinical progression in PD. *GBA* mutations are thought to increase the risk of PD predominantly through perturbations in the lysosome-autophagy system—a system important in α-synuclein clearance. Furthermore, glucocerebrosidase and α-synuclein have been shown to interact directly in vitro and to coexist in Lewy bodies of patients with PD, with a greater proportion of Lewy bodies containing glucocerebrosidase in patients with *GBA* mutations compared with those in patients with PD not carrying a *GBA* variant.^4 9^ It has been speculated that mutant glucocerebrosidase potentiates the aggregation of α-synuclein, and it is therefore feasible that *SNCA* variants such as the rs356219 polymorphism alters the degree to which *GBA* variants predispose to PD pathology and disease progression. It has also been suggested that α-synuclein impedes the transit of glucocerebrosidase from the endoplasmic reticulum to the lysosome, further supporting the idea that the relationship between these two proteins is directly important in the pathogenesis of *GBA*-PD.^3^ The proposed synergistic interaction between variants in the *GBA* gene and *SNCA* gene therefore could also potentially be explained by differences in the degree to which α-synuclein impairs normal glucocerebrosidase processing associated with specific *SNCA* variants.

We acknowledge that our sample size is small, but these preliminary observations raise the possibility that *GBA* variants and the G/G *SNCA* rs356219 polymorphism synergistically alter motor progression in PD. In this study we have considered the time from diagnosis to the development of important clinical milestones in PD. The time to diagnosis may vary between patients, so it should be recognised that this does not necessarily reflect disease duration. Time from disease onset to the development of clinical milestones may be more representative of disease duration, but onset is very difficult to ascertain accurately, given that many patients experience prodromal symptoms that are not initially attributed to PD.

There is a degree of genotype–phenotype correlation in *GBA*-PD, with severe mutations accelerating disease course to a greater extent than less severe variants.^10^ However, because our sample size was small, it was not possible to stratify the patients with *GBA*-PD into those with non-severe and severe *GBA* variants, so our *GBA*-PD population was genetically heterogeneous (online supplementary table 2). Mean age at diagnosis was approximately 5 years later in G/G carriers which may potentially have contributed to their accelerated disease course. However, there were no differences in time to dementia or death, and when age at diagnosis was accounted for in the Cox regression model, the accelerated progression to HY3 persisted. Another limitation of our study is that all subjects in the G/G group were male, and though this was accounted for in the Cox regression model and by performing the additional male-only analysis, it would be important to investigate the relationship between these two genetic risk factors in a large cohort, to reduce the effect of such confounders and to allow for the stratification of G/G carriers into those with severe and those with mild *GBA* variants.
