# Supplementary material for: A common polymorphism in SNCA is associated with accelerated motor decline in GBA-Parkinson’s disease
Source: J Neurol Neurosurg Psychiatry. 2020 Apr 2;91(6):673–4. doi: 10.1136/jnnp-2019-322210 (PMC7279193; doi:10.1136/jnnp-2019-322210)
Supplement: Supplementary data [file jnnp-2019-322210supp001.pdf]

Supplementary Information

	G/G	G/A and A/A	P-value
**Number**	7	13	-
**Gender M:F**	7:0	7:6	0.032
**Age at diagnosis (SD)**	72.4 years (10.3)	75.5 years (9.3)	0.297
**Years in education (SD)**	9.7 years (1.1)	10.6 years (2.8)	0.428
**Total UPDRS at baseline (SD)**	48.8 (15.7)	44.1 (14.0)	0.5
**Motor UPDRS at baseline (SD)**	21.9 (13.0)	30.8 (9.9)	0.825
**Hoehn & Yahr stage at baseline (SD)**	2.1 (0.6)	2.3 (0.6)	0.445
**MMSE at baseline (SD)**	27.6 (1.5)	27.2 (1.9)	0.624
**BDI at baseline (SD)**	7.9 (5.5)	7.4 (5.6)	0.858
**Baseline levodopa equivalent dose (SD)**	188.6 mg (201.0)	185.4mg (213.3)	0.974

Supplementary Table 1 – Patient demographics.

**Patient Number**	***GBA* Variant**	***SNCA* Genotype**
**1**	E326K	G/G
**2**	T369M	G/G
**3**	N370S*	G/G
**4**	c. 762 - 18 T>A	G/G
**5**	L444P*	G/G
**6**	T369M	G/G
**7**	c. 762 - 18 T>A	G/G
**8**	T369M	A/G
**9**	c. 762 - 18 T>A	A/G
**10**	E326K	A/G
**11**	T369M	A/G
**12**	E326K	A/G
**13**	T369M	A/G
**14**	R257Q*	A/G
**15**	E326K	A/A
**16**	R48W*	A/A
**17**	L119L	A/A
**18**	c. 762 - 18 T>A	A/A
**19**	E326K	A/A
**20**	N462K*	A/A

Supplementary Table 2 – *GBA* Variants and *SNCA* rs356219 carrier status in all subjects.

Asterixes indicate variants previously associated with Gaucher disease.

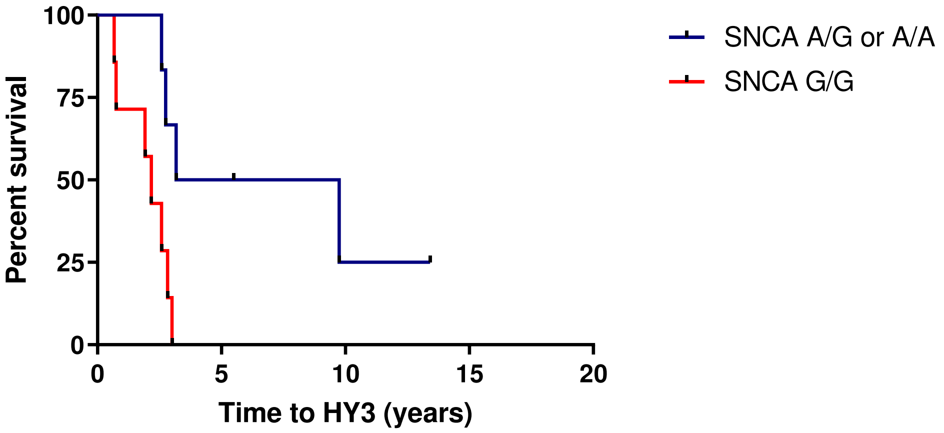

Supplementary figure 1 – Survival analyses in *GBA1*-PD patients comparing those with high- and low-risk variants in the SNCA rs356219 polymorphism. Kaplan-Meier curves for time to postural instability. To control for sex as a potential confounder, female patients were excluded, as all subjects in the *SNCA* G/G group were male. Abbreviations: HY3 = Hoehn and Yahr stage three.

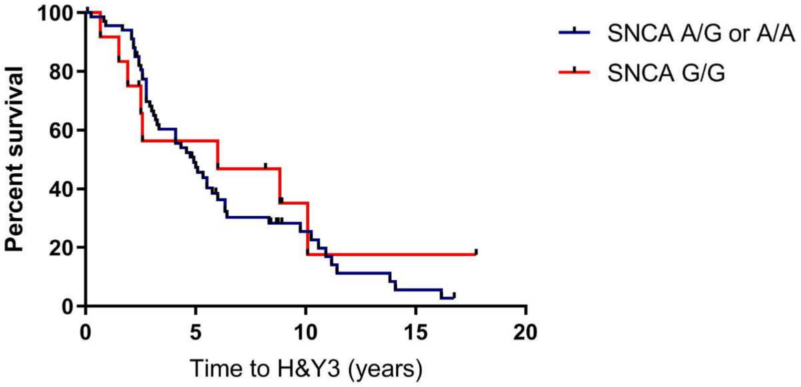

Supplementary Figure 2 – Time to postural instability in PD patients without *GBA* variants, comparing those with high- and low-risk variants in the *SNCA* rs356219 polymorphism. Kaplan-Meier curve for time to postural instability in patients, comparing those carrying the G/G *SNCA* rs356219 variant to A carriers at this locus. Abbreviations: H&Y3 = Hoehn and Yahr stage three.
